# Lectures on Surgical Pathology, Delivered at the Royal College of Surgeons of England

**Published:** 1853

**Authors:** 


ART. IX.—*Lectures on Surgical Pathology*, delivered at the Royal College of Surgeons of England. By JAMES PAGET, F. R. S., &c. Philadelphia, Lindsay & Blakiston, 1854. 8vo, pp. 699.

The name of the author of these Lectures has been rendered familiar to our readers by its association with a manual of Physiology, by Dr. Kirkes, of which Mr. Paget's labors form an important part. He ranks among the most original and philosophical minds in England devoted to the advancement of Medical Science, and in the work before us has developed some highly interesting principles in Physiology and Pathology. As its title implies, it is a work on the Pathology, and not the practice of Surgery, and the reader will be disappointed if he expects to find in it anything in relation to the treatment of the various diseases included in its table of contents. But a clearer or more accurate account of all that pertains to their pathology, we venture to affirm, he will nowhere find, than that contained in these lectures.

As introductory to the morbid changes in the animal body, Mr. Paget considers the process of nutrition, and the circumstances necessary to its healthy performance; and he begins by pointing out what he regards as the difference between development and growth, two terms frequentty used as synonymous.

Growth is mere increase; development is the acquiring of new forms and structures, adapting the individual to higher conditions of existence. The fœtus grows during its intra-uterine life, but it also undergoes rapid development, while after birth there is little development, but constant growth up to maturity. Its muscles are developed out of neucleated cells, and its intestines, stomach, liver, and pancreas, out of a single digestive cavity in its embryonic condition, and so, from a single body, the kidneys, testes of the male, and the ovaria of the female, are developed during the earlier months of fœtal life, and when developed, these organs go on to increase in size. The brain in the first months of the embryo consists of a single lobe, the anterior, from which the middle and posterior lobes afterwards grow out as off-shoots. Arrest of development or arrest of growth may take place, and either will result in enfeeblement of intellect. In one case, there is a deficiency in the parts of the brain; in the other, all the parts are present, but too diminutive in size. The first is an instance of imperfect development, the second of imperfect growth. An earlier arrest of development produces acephalous monsters, as its suspension at the fifth month causes idiocy. And in like manner as to the heart; growth may continue, probably does generally continue, up to an advanced period of life, but development is completed during intra-uterine existence; and when this process is arrested there is malformation, which takes the shape of one cavity, the septum being absent, or two cavaties, with a septum incomplete, according to the period at which the arrest occurred.

We may cite another illustration. Most of our readers, doubtless, have read the story of O'Byrne, the giant, whose skeleton the great John Hunter was so careful to secure for the Museum of the Royal College of Surgeons. The poor fellow, it is said, was suspicious that the old anatomist was covetous of his huge bones, and so engaged a friend to carry his body out after he was dead and bury it in the sea. But Hunter was not to be eluded, and the skeleton, in all its vast proportions, adorns the museum of the College. It is eight feet in height. By the side of it is suspended the skeleton of a female dwarf, measuring only twenty inches. Development was equally complete in these individuals, for not a part belonging to the frame of the giant is wanting in the skeleton of the dwarf, but the disparity of growth is eminently striking.

In growth no change of form or composition occurs; parts only increase in size; but in development there is improvement in quality, a change to a higher state of form or composition.

Nutrition is indispensable to the preservation of the body in its integrity during the whole of life, the organization of which is the subject of continual deterioration and wear and tear. It is worn out by exercise, and its tissues are subject to a natural decay.

"From the first of these, the wearing-out of parts by exercise, it is probable that no tissue or part enjoys immunity. For although, in all the passive apparatus of the body—the joints, bones, ligaments, elastic vessels, and the like—much of the beauty of their construction consists in the means applied to diminish the effects of the friction, and the various pressures and stretchings to which they are subject, yet, in enduring these at all, they must be impaired, and, in the course of years, must need renewal. Doubtless, however, the waste of these parts by exercise is much less than that of the more active organs, such as the muscles, and, perhaps, the nervous system. With regard to the muscles, it is clear that chemical decomposition and consumption of their substance attend their continued action. Such action is always followed by the increased discharge of urea, carbonic acid and water. The researches of Helmholtz show, that the muscles themselves, after long repeated contractions, are changed in chemical composition; and those of G. Liebig, have detected and measured the formation of carbonic acid in them during similar contractions."

But apart from the exhausting effects of exercise which hastens the disintegration of the tissues, there is a natural term of existence to each elemental part of the body; and at the end of this period it degenerates and is absorbed, or dies, and is cast out. We see the proofs of this in the deciduous teeth, and in the hair, which after an appointed time are removed by the process of absorption, spontaneously, independently of exercise, as the body itself, at a later term, yields to the same general law. And so the ordinary course of all the tissues of the body, after the attainment of their perfect state, is to remain in that state for a time, and then to degenerate and die, and being cast out or absorbed, make way for their successors.

"It appears moreover very probable, that the length of life which each part is to enjoy is fixed and determinate, though of course in some degree, subject to accidents, which may shorten it, as sickness may prevent death through mere old age; and subject to the expenditure of life in the exercise of function. I do not mean that we can assign, as it is popularly supposed we can, the time that all our parts will last; nor is it likely that all parts are made to last an equal time, and then to be changed. The bones, for instance, when once completely formed, must last longer than the muscles and other softer tissues. But, when we see that the life of certain parts is of determined length, whether they be used or not, we may assume, from analogy, the same of nearly all.

For instance, the deciduous human teeth have an appointed duration of life: not, indeed, exactly the same in all persons, yet, on the whole, fixed and determinate. So have the deciduous teeth of other animals. And, in all those numerous instances of periodical moulting, of shedding of the antlers, of the entire desquamation of serpents, and of the change of plumage in birds, and of the hair in mammalia; what means all this, but that these organs live their severally appointed times, degenerate, die, are cast away, and in due time are replaced by others; which, in their turn, are to be developed to perfection, to live their life in the mature state, and to be cast off? We may discern the same laws of life in some elementary structures; for example, in the blood-corpuscles, of which a first set, formed from embryo-cells, disappear at a certain period in the life of the embryo, being replaced and superseded by a second set formed from lymph-corpuscles. And in these, also, we may see an example of the length of life of elemental parts being determined, in some measure, by their activity in function; for if the development of the tadpole be retarded, by keeping it in a cold, dark place, and if, in this condition; the function of the first set of blood-corpuscles be slowly and imperfectly discharged, they will remain unchanged for even many weeks longer than usual: their individual life will be thus prolonged, and the development of the corpuscles of the second set will be, for the same time postponed."

The conditions necessary to the healthy nutrition of a part are, a right state and composition of the blood, a regular and not far distant supply of such blood, healthy nervous influence, and a natural state of the part to be maintained.

It is only of late that the minds of medical men have been duly turned to those changes to which the blood is subject, and which give origin to various disturbances of the system. There is ordinarily a remarkable persisttance in the character of this fluid, notwithstanding the diversity of materials put into it, and the numerous secretions separated from it, insomuch that throughout life it retains, in each person, certain qualities as peculiar as his outer features. But, remarks Mr. Paget:

"Notwithstanding its possession of the capacity of maintenance, the blood is subject to various diseases, in consequence of which the nutrition of one or more tissues is disordered. The researches of modern chemistry have detected some of these changes; finding excesses or deficiencies of some of the chief constituents of the blood, and detecting in it some of the materials introduced from without. But a far greater number of the morbid conditions of the blood consists in changes from the discovery of which the acutist chemistry seems yet far distant, and for the illustration and discussion of which we cannot adopt the facts, though we may adopt the language and the analogies, of chemistry. It is in such diseases as these that we can best discern how nice is that refinement of mutual influence, how exact and constant that adaptation, between the blood and tissues, on which health depends."

The necessity of the second condition is shown by the effect of mechanical obstructions to the circulation, which is often the loss of vitality in a part. Thus an injury done to the femoral or brachial artery may lead to gangrene of the extremity, as excessive loss of blood has been known to be followed by sloughing of the nose, instances of which are cited by Mr. Paget. The following is in point:

"And, lastly, let me refer to two specimens, which are as interesting in the history of surgery as in pathology. One is a tibia and fibula, the lower ends of which, together with the whole foot, perished in consequence of the obstruction of the circulation by an aneurism in the ham. It is an Hunterian specimen in the College Museum (No. 710); and surely we may imagine that sometimes Mr. Hunter would contemplate it with pride, to think how rare such things would be in after times; for here is a strong contrast: the limb of a man who once had an aneurism, like the one which in the former case was so destructive, and on whom Hunter was permitted to confer fifty years of healthy life by his operation of tying the artery at a distance from the diseased part. The Museum of St. Bartholomew's owes this rare specimen and most interesting relic to the zeal of my colleague, Mr. Wormald. The patient was the fourth on whom Mr. Hunter performed his operation. He was 36 years old at the time; and although the tumor was not large, yet the whole leg was swollen, the veins were turgid, and he was exhausted, and in such bad health, that the case seemed desperate; but he recovered, and lived, as I have said, fifty years. The artery was tied in the sheath of the triceps muscle; and in this operation, for the first time, Mr. Hunter did not include the vein in the ligature. He thus diminished exeedingly the danger of the defective supply of arterial blood. The preparation shows the whole length of the artery obliterated from the origin of the profunda, to that of the anterior tibial, and the aneurismal sac, even after fifty years, not yet removed, but remaining as a hard mass like an olive."

Mr. Paget insists upon the influence of the nervous system upon the function of nutrition, and indeed the proof of the fact is ample. The control of the mind over the body, its agency in inducing and curing disease, do we not witness examples of it every day? Mr. P. relates some which may stand with the most remarkable, as, for instance, the following:—

"Mr. Lawrence removed, several years ago, a fatty tumor from a woman's shoulder; and, when all was healed, she took it into her head that it was a cancer, and would return. Accordingly, when by accident I saw her a month afterwards she was in a workhouse, and had a large and firm painful tumor in her breast, which, I believe, would have been removed, but that its nature was obscure, and her general health was not good. Again, some months afterwards, she became my patient at the Finsbury Dispensary: her health was much improved, but the hard lump in her breast existed still, as large as an egg, and just like a portion of indurated mammary gland. Having heard all the account of it, and how her mind constantly dwelt in fear of cancer, I made bold to assure her, by all that was certain, that the cancer, as she supposed it, would go away: and it did become very much smaller without any help from medicine. As it had come under the influence of fear, so it very nearly disappeared under that of confidence. But I lost sight of her before the removal of the tumor was complete."

The last condition essential to healthy nutrition, is a healthy state of the part to be nourished, upon which Mr. Paget remarks as follows:

"This is, indeed, involved in the very idea of the assimilation which is accomplished in the formative process, wherein the materials are supposed to be made like to the structures among which they are deposited: for unless the type be good, the anti-type cannot be.

In a part which was originally well formed, and with which the three conditions of nutrition already illustrated have been always present, this fourth condition will probably be never wanting; for the part will not of itself deflect from the normal state. But when any part, or any constituent of the blood, has been injured or diseased, its unhealthy state will interfere with its nutrition long after the immediate effects of the injury or disease have passed away. Just as, in healthy parts, the formative process exactly assimilates the new materials to the old, so does it in diseased parts; the new-formed blood and tissues take the likeness of the old ones in all their peculiarities whether normal or abnormal; and hence the healthy state of the part to be nourished may be said to be essential to the healthy process of nutrition.

The exactness of assimilation accomplished by the formative process in healthy parts has been already, in some measure, illustrated, as preserving through life certain characteristic differences, even in the several parts of one organ; preserving, also, all those peculiarities of structure and of action, which form the proper features, and indicate the temperament of the individual. In these, and in a thousand similar instances, the precision of assimilation in the formative process is perfect and absolute, except in so far as it admits of a very gradual alteration of the parts, in conformity with the law of change in advancing years.

Nor is there less of exactness in the assimilation of which a part that has been diseased is the seat. For, after any injury or disease, by which the structure of a part is impaired, we find the altered structure,—whether an induration, a cicatrix, or any other,—as it were, perpetuated by assimilation. It is not that an unhealthy process continues: the result is due to the process of exact assimilation operating in a part of which the structure has been changed: the same process which once preserved the healthy state, maintains now the diseased one. Thus, a scar or a diseased spot may grow and assimilate as its healthy neighbo:s do. The scar of the child, when once completely formed, commonly grows as the body does, at the same rate, and according to the same general rule; so that a scar which the child might have said was as long as his own forefinger, will still be as long as his forefinger when he grows to be a man.

Yet through this increase and persistence of the morbid structure be the general and larger rule, another within it is to be remembered; namely, that in these structures there is usually (especially in youth) a tendency towards the healthy state. Hence, cicatrices, after long endurance, and even much increase, may, as it is said, wear out; and thicknings and induration of parts may give way, and all become again pliant and elastic.

The maintenance of morbid structures is so familiar a fact, that not only its wonder, but its significance, seem to be too much overlooked. What we see in scars and thickenings of parts appears to be only an example of a very large class of cases; for this exactness by which the formative process in a part maintains the change once produced by disease, offers a reasonable explanation of the fact that certain diseases usually occur only once in the same body. The poison of small-pox, or of scarlet fever, being, for example, once inserted, soon, by multiplication or otherwise, affects the whole of the blood; alters its whole composition: the disease, in a definite form and order, pursues its course; and, finally, the blood recovers, to all appearance, its former state. Yet it is not as it was: for now the same material, the same variolous poison, will not produce the same effect upon it; and the alteration thus made in the blood or the tissues is made once for all: for, commonly, through all after life, the formative process assimilates, and never deviates from, the altered type, but reproduces materials exactly like those altered by the disease; the new ones, therefore, like the old, are incapable of alteration by the same poison, and the individual is safe from the danger of infection."

A subject of interesting inquiry is involved in the difference to be perceived between what Mr. Paget calls 'nutritive reproduction and nutritive repetition,' and which he thus illustrates by the teeth and the blood:—

"In our own case, as the deciduous tooth is being developed, a part of its productive capsule is detached, and serves as a germ for the formation of the second tooth; in which second tooth, therefore, the first may be said to be reproduced, in the same sense as that in which we speak of the organs by which new individuals are formed, as the reproductive organs. But in the shark, in which we see row after row of teeth succeeding each other, the row behind is not formed from germs derived from the row before: the front row is simply repeated in the second one, the second in the third, and so on.

It is the same in the blood. The new blood-corpuscles, that are being constantly formed for the renovation of the blood, are not developed from germs given off from the old ones; neither are they formed by any assimilative force exercised by the old ones. By watching the stages of their construction, we may see that the development of each is an independent repetition of the process by which the first were formed. And so with the successive developments of ova and epithelial cells, and many others; each is developed independently of the rest, and each repeats the changes through which its predecessor passed."

Hypertrophy is a form of nutrition, and though it may give rise to morbid phenomena is not itself a morbid process. The cuticle of the hand is hypertrophied in the laborer, as the muscles of the leg are in the rope-dancer, and those of the arm in the blacksmith. The process is the same as in the hypertrophy of the heart, which results from disease of its valves or of some other part :—

"In a great majority of cases, the hypertrophy of muscles, whether voluntary or involuntary, is the consequence of an increased obstacle to their ordinary action. Against this obstacle they exert extraordinary force, and this induces, indirectly, extraordinary formation of their tissue. Frequent action of muscles, unless it be also forcible, does not produce hypertrophy. As Mr. Humphrey says, the heart, though it may act with unusual frequency for years, yet does not in these cases grow larger; and the muscles of the hands are not generally so large in mechanics who use great celerity of action, as in those who work with great force. But action of muscles, if it be at once frequent and forcible, may produce hypertrophy, even though the action be unhealthy. This appears to be the case with the bladders of some children, who suffer with frequent and very painful micturition, and nearly all the signs of calculus, but in whom no calculus exists. The bladder in such children is found, after death, exceedingly hypertrophied, and there may be no other disease whatever of the urinary organs. Dr. Golding Bird has shown that phymosis, by obstructing the free exit of urine, may give rise to these signs and to extreme hypertrophy of the bladder; but in some cases it appears certain that hypertrophy may occur without either phymosis, calculus, stricture, or any similar obstruction. It was so in a case illustrated in the Museum of St. Bartholomew (xxvii. 14), in a child four years old, who had suffered intensely with signs of stone in the bladder, but in whom no stone existed; no disease of the urinary organs could be found, except this hypertrophy of the muscular coat of the bladder. An exactly similar case has been recently under Mr. Stanley's care, in which, after exceeding irritability of the bladder, the enlargement of its muscular coat appeared the only change.

In such cases, the too frequent and strong action of the bladder, though irritable and unhealthy, seems alone to give rise to hypertrophy of the fibres. It is, however, possible that the change may be due to narrowing of the urethra, by muscular action. If, for example, the compressors of the urethra, instead of relaxing when the muscular coat of the bladder and the abdominal muscles are contracting, were to contract with them, the obstacle they would produce in the urethra would soon engender hypertrophy of the bladder.

Hunter, whose ingenuity was ever tempting on his intellect and industry, asked himself whether the hypertrophy of the heart were accomplished by the addition of new fibres, or by the enlargement of those that already exist. This question could hardly be determined without more microscropic aid than Hunter had at his command. But if we may believe (and there can be no doubt we may) that hypertrophy is, in this respect also, exactly similar to common growth, the question set by Hunter has been answered by Harting, with whom, on this point, Kolliker agrees. He has shown that, in the growth of striped muscles, there is no multiplication, no numerical increase, of the fibres, but an enlargement of them with addition to the number of the fibrils."

On the opposite process—atrophy, Mr. Paget has some interesting remarks, a portion of which we quote. We have seen that the body is the seat of ceaseless degeneration, parts becoming effete, useless, injurious if retained, and so cast out, after a time. As years accumulate, this degeneration becomes more rapid than the supply of new materials, and senile atrophy begins. The powers which prevailed over the waste of the organism in infancy and youth, and maintained the balance in vigorous manhood, have failed, and there is henceforward the decay of old age.

"All the expressions usually employed about these changes imply that they are not regarded as the results of disease: nor should they be; they are, or may be, completely normal; and were it not that the forces which are efficient in degeneration are, probably, very different from those which actuate the formative processes, we might justly call the degeneration of advanced age another normal method of nutrition. For, to degenerate and die is as normal as to be developed and live: the expansion of growth, and the full strength of manhood, are not more natural than the decay and feebleness of a timely old age; not more natural, because not more in accordance with constant laws, as observed in ordinary conditions. As the development of the whole being, and of every element of its tissues, is according to certain laws, so is the whole process regulated, by which all that has life will, as of its own workings, cease to live. The definition of life that Bichat gave is, in this view, as untrue as it is illogical. Life is so far from being the "the sum of the functions that resist death," that it is a constant part of the history of life that its exercise leads naturally to decay, and through decay to death.

Of the manner in which this decay or degeneration of organisms ensues we know but little. Till within the last few years the subject of degenerations was scarcely pursued: and, even of late, the inquiries, which ought to range over the whole field of living nature, have been almost exclusively limited to the human body. The study of development has always had precedence in the choice of all the best workers in physiological science. They who have devoted many years of laborious thought and observation to the study of the changes by which the living being is developed from rudiment to perfection, have given fewer hours to the investigation of those by which, from that perfection, it naturally descends into decay and death. Almost the only essays at a general illustration of the subject have issued in the ridiculous notion that, as the body grows old, so it retrogrades into a lower station in the scale of animal creation. The flattened cornea is supposed to degrade the old man to the level of the fish; while the *arcus senilis*, by a fancied correspondence with an osseous sclerotic ring, maintains him in the eminence of a bird: his dry thick cuticle makes him like the pachydermata; and his shrivelled spleen approximates him to the humility of the mollusc. One can only commend such day-dreams to the modern supporters of the doctrine of transmutation of species; and they might, indeed, form an appropriate supplement to their scheme, if they would maintain that, in these latter days, our species is destined to degenerate into lower and yet lower forms, descending through the grades by which, in bygone times, it ascended to its climax in humanity."

All the tissues of the body seem liable, though in different degrees, to a degeneration connected with defective nutrition. It is often witnessed in the muscles, in the great organs, and occasionally in the blood vessels, the bones, &c. Many examples are cited by Mr. Paget, of which we can afford room for but a single one:—

"The last example of atrophy of which I will speak, is that which is manifested in the arcus senilis,—the dim grayish-white arches or ellipse seen near the borders of the cornea in so many old persons. Its nature, as a true fatty degeneration, consisting in the accumulation of minute oil-drops in the proper tissue of the cornea, was discovered and is fully described by Mr. Canton. By his and others' investigations, it has also acquired a larger interest, in being found the frequent concomitant and sign of more widely extended degenerations that are not within sight during life. Thus, it is commonly associated with fatty or calcareous degeneration of the ophthalmic artery; with fatty degeneration of the muscles of the eyeball; and, especially in old persons, with fatty degeneration of the heart and many other organs. In short, the arcus senilis seems to be, on the whole, the best indication that has been yet found of proneness to an extensive or general fatty degeneration of the tissues. It is not, indeed, an infallible sign thereof; for there are cases in which it exists with clear evidences of vigor in the nutrition of the rest of the body; and there are others in which its early occurrence is due to defective nutrition consequent on purely local causes, such as inflammatory affections of the choroid, or other parts of the eye: but, allowing for these exceptions, it appears to be the surest, as well as the most visible, sign and measures of those primary degenerations which it has been the chief object of the last two lectures to describe."

Besides the topics briefly noticed in this article, some of the more interesting subjects discussed in these lectures are inflammation, repair and reproduction, tumors, cancer, and tubercle; and the reader in quest of precise and full nformation on these points will no where find more to epay research than in this work.

